# First Report of Marek’s Disease Virus in Commercial Turkeys in Slovenia

**DOI:** 10.3390/ani14020250

**Published:** 2024-01-12

**Authors:** Zoran Žlabravec, Brigita Slavec, Ema Rožmanec, Saša Koprivec, Alenka Dovč, Olga Zorman Rojs

**Affiliations:** 1 Institute of Poultry, Birds, Small Mammals, and Reptiles, Faculty of Veterinary Medicine, University of Ljubljana, Gerbičeva ulica 60, 1000 Ljubljana, Slovenia; zoran.zlabravec@vf.uni-lj.si (Z.Ž.);; 2 Veterinarska Ambulanta PP, d.o.o., Potrčeva cesta 10, 2250 Ptuj, Slovenia; 3 Institute of Preclinical Sciences, Faculty of Veterinary Medicine, University of Ljubljana, Gerbičeva ulica 60, 1000 Ljubljana, Slovenia

**Keywords:** Marek’s disease, turkey, Mardivirus gallidalpha 2, *meq* gene, molecular characterization, Slovenia

## Abstract

**Simple Summary:**

Marek’s disease (MD) is caused by herpesvirus and is a common disease of chickens, usually characterized by tumors in various tissues. Lately, numerous studies have also shown an increasing incidence of the disease in turkeys. This report describes various clinical signs and pathological changes in three different flocks of commercial turkeys in Slovenia. Molecular analysis confirmed the presence of identical Marek’s disease virus (MDV) strains in all three cases and showed that the MDV detected is most similar to the MDV detected in chickens in Tunisia.

**Abstract:**

Marek’s disease (MD), caused by *Mardivirus gallidalpha 2* (GaAHV-2), also known as MD virus (MDV), is a lymphoproliferative disease that primarily affects chickens. Recently, MDV has been detected in lymphomatous tumors in turkeys in various countries. Between 2021 and 2023, three cases ranging from no to severe clinical disorders (depression, lameness, and increased mortality) occurred in commercial turkey flocks in Slovenia. In all cases, MDV was detected by PCR in DNA samples extracted from organs developing tumor infiltrations. Sequencing and phylogenetic analysis of the *meq* gene revealed that the GaAHV-2 detected has molecular features of a very virulent pathotype and genetic similarity with GaAHV-2 detected in chickens in Tunisia. This is the first report of MDV in commercial turkeys in Slovenia.

## 1. Introduction

Marek’s disease (MD) is one of the most important neoplastic and immunosuppressive diseases, and it is responsible for great economic loss in the poultry industry worldwide due to decreased productivity, increased morbidity and mortality, and condemnation at slaughter. The primary natural host is chickens, although MD has also been sporadically reported in turkeys, quail, pheasants, and some species of geese and ducks. The disease is characterized by the onset of lymphoid tumors in various organs, immunosuppression, and paralysis [1]. The causative agent is *Mardivirus gallidalpha 2* (GaAHV-2), previously known as Marek’s disease virus (MDV), a member of the genus *Mardivirus* of the subfamily *Alphaherpesvirinae*, the family *Orthoherpesviridae*, and the order *Herpesvirales*. The genus *Mardivirus* also includes *Mardivirus anatidalpha 1* (AnAHV-1), *Mardivirus columbidaalpha 1* (CoAHV-1), *Mardivirus Spheniscidalpha 1* (SpAHV-1), *Mardivirus gallidalpha 3* (GaAHV-3), and *Mardivirus meleagridalpha 1* (MeAHV-1; turkey herpesvirus—HVT) [2]. HVT is endemic and ubiquitous in domestic and wild turkeys. In chickens, the virus has also become ubiquitous because of its widespread use as a vaccine against MD. Even though it gives protection against MD in chickens, naturally circulating HVT does not appear to protect turkeys against MD [3]. Virulence or oncogenicity is only associated with GaAHV-2, for which wide variation in its pathogenic potential, from nearly avirulent to maximally virulent, is recognized [4]. The current pathotype classification of GaAHV-2 was performed in chickens and includes mild (m)MDV, virulent (v)MDV, very virulent (vv)MDV, and very virulent plus (vv+) MDV [5]. MDV has a dsDNA genome of approximately 160 to 180 kb that encodes more than 200 genes. The genome of MDV encodes *Marek’s EcoRI-Q* (*meq*), which is known to be a transcription factor involved in the regulation of the cell division cycle [6]. It is one of the principal oncogenes of MDV and also contributes to immunosuppression [1].

Reports on MD-induced tumors in turkeys are rare, but they have been increasing over the past decades. MD has been described in commercial turkeys from the Netherlands [7], France [8], Germany [9], Israel [10], and the UK [3,11]. Most recently, tumors associated with GaAHV-2 infection were confirmed in commercial free-range turkeys in Italy [12] and backyard turkeys from the US [13] and Turkey [14]. The clinical signs associated with MD in turkeys are non-specific, and they mostly appear between 12 and 30 weeks of age. Infected birds show stunted growth, apathy, and lameness [13,15]. In one report, mortality up to 60% at 20 weeks was reported [8]. At necropsy, lymphoid tumors in the liver, spleen, and kidney have been seen, although pathological changes in nerves have seldom been observed. Generally, the diagnosis of MD in turkeys is based on histopathology and molecular identification of MDV [15].

This study reports clinical signs and lesions, including visceral tumors with subsequent molecular characterization of the GaAHV-2 *meq* region, detected in three cases of MD that occurred in commercial turkey flocks in Slovenia.

## 2. Materials and Methods

### 2.1. Case History

The first case (flock 1; case number 39/21) was detected in a flock of 20-week-old commercial toms in January 2021 at a slaughter plant. During the veterinary inspection after evisceration, white nodules of varying sizes were observed in different visceral organs in 13 (1.23%) out of 1056 turkey carcasses. Lesions were predominantly seen in the liver, spleen, and kidney. Samples of the liver, spleen, heart, intestine, pancreas, and kidney were collected by the authorities and submitted to the Institute of Poultry, Birds, Small Mammals, and Reptiles for further investigation. The remaining 894 turkeys from the same flock were slaughtered 11 days later, and neoplastic lesions were found in 10 (1.12%) out of 894 carcasses. Before the first slaughter and during the period between both slaughters, no specific clinical signs were observed and no elevated daily mortality was recorded.

The second case (flock 2; case number 1247/22) occurred in a commercial flock of 17-week-old toms in July 2022. Clinical signs reported 20 days after the hens were removed from the barn for slaughter included increased mortality, lameness, and depression. The turkeys affected were apathetic, pale, and almost unable to move. Gross examination was performed following a routine procedure by a field veterinarian. Enlarged livers, spleens, and kidneys with multifocal nodular structures of varying sizes were observed. Nodular structures were also seen in the heart and lungs. Higher mortality was recorded from 16 weeks of age until slaughter (at 21 weeks); during this period, 9.89% of the birds died. At the slaughter plant, neoplastic lesions were found in 441 (14.04%) out of 3142 carcasses.

The third case (flock 3; case number 873/23) occurred in 15-week-old toms in May 2023. Five days after the hens were removed from the barn for slaughter, clinical signs such as depression, lameness, weakness, and increased mortality were noticed. At necropsy, in addition to dehydration and cachexia, nodular structures were seen in various visceral organs. Due to daily increasing mortality—from 105 days to 121 days of age, 5.62% of birds died—it was decided to slaughter the toms earlier than planned. At the slaughter plant, 342 (11.05%) out of 3099 carcasses were confiscated, mainly due to neoplastic lesions seen on various organs.

All of the flocks affected had been reared in fully enclosed and insulated facilities, and no other poultry species were present on the farms. On all farms, the turkey breed was B.U.T. 6, and birds of both sexes were kept in the same facilities until slaughtering the hens at around 14 to 15 weeks of age. The birds from all the flocks affected were not vaccinated against MD.

### 2.2. Pathology/Histopathology

The organs submitted were grossly examined, and tissue specimens (liver, spleen, heart, intestine, pancreas, and kidneys from the first case; liver, spleen, kidneys, heart, and lungs from the second and third cases) were fixed in a 10% neutral buffered formalin solution. For histopathology, the tissues were embedded in paraffin, sectioned at 4 µm, mounted on glass slides, and stained with hematoxylin and eosin (H&E) using the routine method.

### 2.3. DNA Extraction from Tissue Samples

Selected tumor-bearing organs (liver, heart, spleen, and kidney) from two turkeys from each flock were used for the genomic DNA extraction. Swabs of pooled homogenized tissue from each animal were vortexed in 2 mL phosphate-buffered saline for 1 min, and the supernatant was stored for nucleic acid extraction. Total DNA and RNA were extracted from 140 μL of the supernatant with the QIAamp Viral RNA Mini Kit (Qiagen, Hilden, Germany) according to the manufacturer’s instructions.

### 2.4. Detection of Viruses by PCR

#### 2.4.1. Detection of MDV by PCR Assay

For amplification of the full-length *meq* gene of the GaAHV-2 family, the primers and cycling conditions described by Mescolini et al. [16] were used. We prepared 20 µL of PCR reaction using 10 µL of 2X DreamTaq Green PCR Master Mix (Thermo Scientific, Dreieich, Germany), 1 µM of each primer, 2 µL of isolated DNA, and deionized water. The cycling parameters included an initial denaturation step at 95 °C for 5 min, followed by 35 cycles at 95 °C for 1 min, 58 °C for 1 min, 72 °C for 90 s, and a final extension step at 72 °C for 10 min. Appropriate positive (positive field strain) and negative (phosphate-buffered saline, PBS) controls were used. PCR products were loaded onto 1.8% agarose gel (Sigma-Aldrich, St. Louis, MO, USA), and electrophoresis was performed at 130 V for 30 min. The gel was stained with ethidium bromide, and bands were visualized using the Bio-Rad Gel Doc 2000 Imaging System, on Quantity One 4.4.0 analysis software. Bands were excised from the gel and purified using the FastGene Gel/PCR Extraction Kit (Nippon Genetics, Duren, Germany.). Additional internal primers based on GaAHV-2 strain HC/0803 (MW531728) were designed and used to obtain a good-quality sequence. The PCR primers used are listed in Table 1. The PCR products were sent to Macrogen Laboratory (Macrogen Europe B.V., Amsterdam, The Netherlands) for Sanger sequencing.

#### 2.4.2. Detection of REV by Real-Time PCR (qPCR) Assay

To exclude the presence of avian reticuloendotheliosis virus (REV) infection, qPCR was used to detect both the *rev envelope* (*env*) gene and the LTR region of proviral DNA. The qPCR primers and probes used are listed in Table 1. A final volume of 15 µL qPCR reaction was prepared using the Kapa probe Fast qPCR kit, containing 20 pmol of each env/LTR primer, 10 pmol of the respective probe, and 2 µL of template DNA. The cycle parameters included an initial incubation step at 50 °C for 2 min and 95 °C for 10 min, followed by 40 cycles of denaturation at 95 °C for 15 s and annealing/elongation at 60 °C for 1 min. Fluorescence was collected at the annealing/elongation step. Appropriate positive (positive field strain) and negative (PBS) controls were applied for each test.

#### 2.4.3. Detection of LPDV by PCR Assay

For diagnosis of lymphoproliferative disease virus (LPDV), a PCR assay used primers that covered a region spanning the *p31* and *ca* genes. The primers are listed in Table 1. The 20 µL PCR reactions included 10 µL 2X DreamTaq Green PCR Master Mix (Thermo Scientific, Europe), 10 pmol of each primer, 2 µL of DNA, and deionized water. The cycle parameters included initial denaturation at 95 °C for 3 min, followed by 35 cycles of denaturation at 95 °C for 30 s, annealing at 54 °C for 30 s, elongation at 68 °C for 1 min, and a final elongation step at 72 °C for 10 min. Appropriate positive (positive field strain) and negative (PBS) controls were applied for each test.

### 2.5. Phylogenetic Analysis

**Figure 1:**
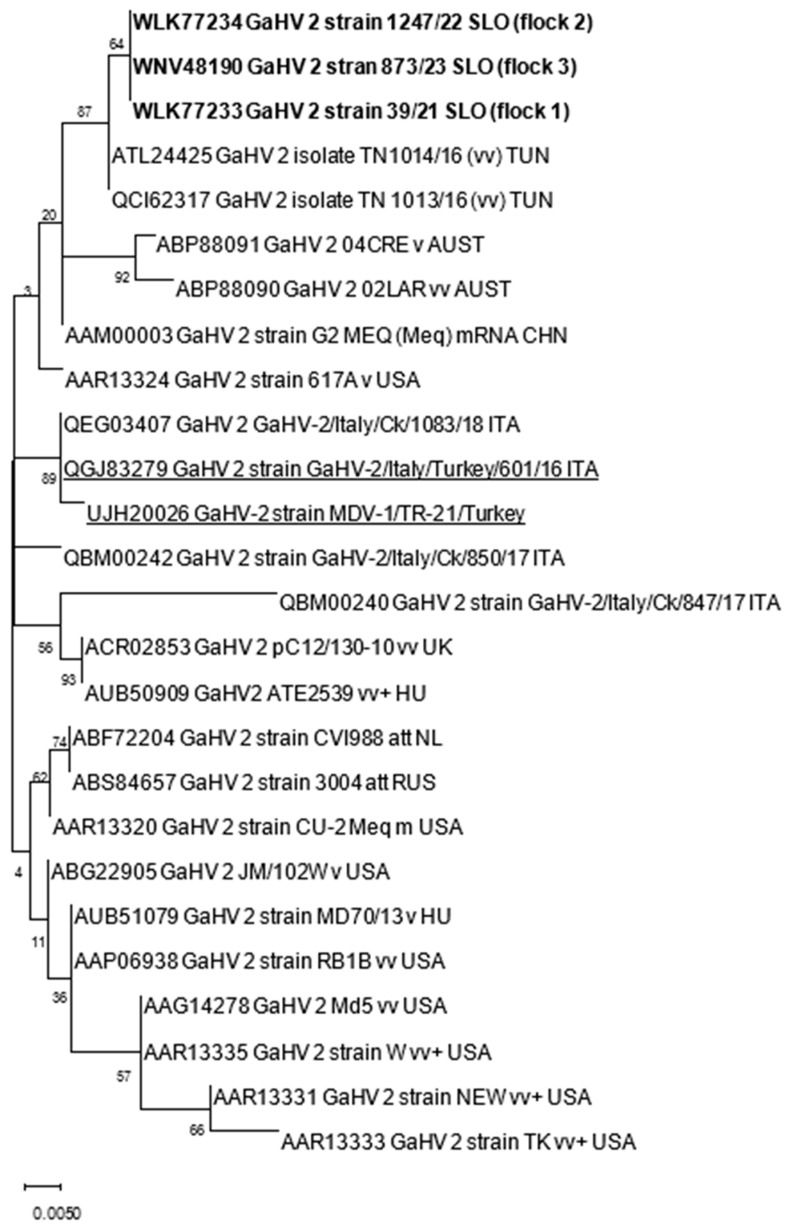
Phylogenetic relationship based on GaAHV-2 *meq* gene amino acid sequences of GaAHV-2 detected in turkeys in Slovenia (text in bold) and other GaAHV-2 derived from the GenBank database. Amino acid sequences obtained from turkeys are additionally underlined.

The nucleotide (nt) and amino acid (aa) sequences obtained were first analyzed with BLAST [19] to identify sequences relevant for further analyses within the NCBI database. Nt and aa alignments were constructed with ClustalW implemented in MEGA X. Phylogenetic analysis was performed using the maximum likelihood method with the Jones–Taylor–Thornton parameter model and 1000 bootstrap replicates by MEGA X [20]. The genetic distances among sequences were calculated using the *p*-distance model (pairwise distance) in MEGA X. The accession numbers of GaAHV-2 sequences used for phylogenetic analysis are included in Table 2 and Figure 1.

## 3. Results

### 3.1. Gross and Histopathological Findings

**Figure 2:**
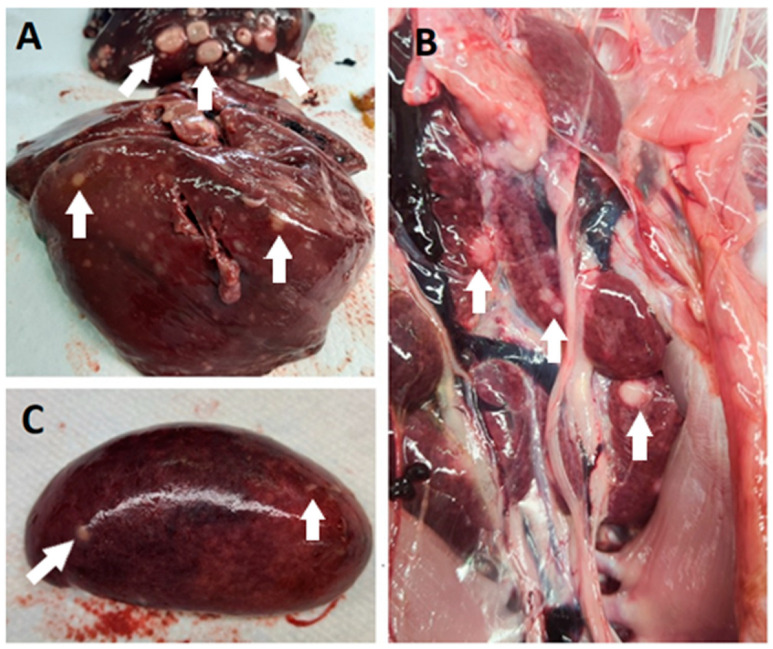
Post-mortem lesions of MD in commercial turkey flocks in Slovenia. Pale white nodules (some nodules are marked with white arrows) were found primarily in the liver (**A**), kidney (**B**), and spleen (**C**).

In all cases, the post-mortem examination revealed white foci and small white nodules in various organs, including the liver, spleen, kidneys, heart, lungs, intestine, and pancreas (Figure 2). In addition, enlarged and dark livers, pale and enlarged spleens, and mottled and pale kidneys were identified in some cases. In the routine histopathological examination, multifocal infiltration of uniform pleomorphic lymphoid cells was identified in the liver, lungs, kidneys, heart, spleen, intestine, and pancreas. In addition, case 39/21 showed multifocal infiltration of heterophils, hepatocyte degeneration, and necrosis in the liver; multifocal hemorrhage in the spleen; and degeneration of acinar cells in the pancreas.

### 3.2. Detection of Viruses by PCR

**Figure 3:**
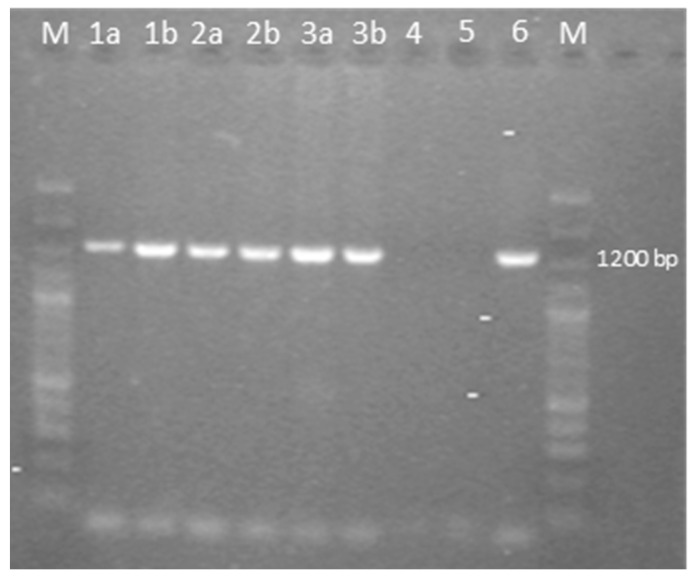
Agarose gel electrophoresis of PCR fragments of the full-length *meq* gene of the GaAHV-2 family, amplified by using the specific primers EcoR-F and EcoR-R. Lane M: 100 bp plus DNA ladder; lane 1a: sample 1 flock 1; lane 1b: sample 2 flock 1; lane 2a: sample 1 flock 2; lane 2b: sample 2 flock 2; lane 3a: sample 1 flock 3; lane 3b: sample 2 flock 3; lane 4: negative control; lane 5: no template control; lane 6: positive control.

The samples analyzed were negative for REV and LPDV and positive at PCR for the GaAHV-2 *meq* gene (Figure 3). As a result of the sequencing of the purified PCR products, the *meq* gene (1020 nt in length) encoding 339 aa was detected. The *meq* gene sequences detected in all three cases (OQ868520/WLK77233, OQ868521/WLK77234, and OR490416/WNV48190) were identical and shared 99.9% nt identity and 99.7% aa identity with the most closely related strain in GenBank, GaAHV-2, detected in chickens in Tunisia (KY113150/ATL24425; Figure 1). Lower nt and aa identities—99.31% and 98.23%, as well as 99.41% and 97.94%—were detected with the GaAHV-2 *meq* gene detected in commercial turkeys in Italy (MN017102/QGJ83279) and backyard turkeys in Turkey (OK322357/UJH20026), respectively.

Four proline-rich repeat regions (PPPPs) were identified in the transactivation domain, together with a PAPP sequence, in which alanine replaced a proline at position 217 (A^217^P). The start of PPPPs in the *meq* gene was based on aa positions 152, 175, 191, and 232.

## 4. Discussion

MDV is one of the most economically important virus-induced transmissible neoplastic diseases of poultry. Despite the widespread use of vaccines and the development of new methods of vaccination, MD still remains a major challenge to chickens’ health, particularly from the continuing increase in virulence of MDV strains [1]. Furthermore, the incidence of MD in other avian species such as turkeys demonstrates the increasing host range and economic significance [15]. However, even in susceptible chickens, infection does not always induce clinical disease, and, in genetically resistant or vaccinated chickens, infection may rarely cause overt disease [1]. Similar findings were noticed in the first case (flock 1), where gross pathological changes were seen at the slaughter plant, even though no clinical symptoms of MD were observed during the fattening cycle. The lymphomatous lesion of MD is one of the most important causes of carcass condemnation in slaughterhouses [30].

On the other hand, in the second case (flock 2) and third case (flock 3), increased mortality, lameness, and depression were observed in the flock of 125-day-old and 105-day-old toms, respectively. Interestingly, 20 days and 5 days earlier, respectively, no gross pathologies were noticed in the hens at the slaughter plant during veterinary inspections. Factors that influence mortality and lesions are virus strain, virus dose and route, maternal antibodies, host genetics, age at exposure, prior infections, environmental factors, stress, and host sex [30]. A study performed in chickens showed that females died earlier and experienced higher losses due to MD than males [31]. This is the opposite of our cases, in which lesions were noticed only or mainly in males. However, there are no data on host sex predisposition in turkeys. One trigger factor that could lead to the clinical form of the disease, mainly in toms, could be stress in a flock induced during the capture of hens for slaughter. Turkeys, similar to chickens, could be persistently infected with MDV, and various factors, including stress, could trigger the onset of the clinical form of Marek’s disease. In both cases, etiological diagnoses were made based on gross lesions and GaAHV-2 *meq* gene detection, as well as negative PCR results for two major differential diagnoses which could be responsible for similar clinical signs and gross lesions to MDV: infection with REV and LPDV. For a more accurate diagnosis, immunohistopathology for the detection of cd3 cells should be performed. However, some studies have shown that MDV-1 can cause B cell tumors in turkeys [32,33], and in rare cases, T cell tumors in turkeys can be caused by REV [34].

Interestingly, despite the detected different clinical onset of disease, phylogenetic analysis of the *meq* gene showed identical sequences in all three cases. The sequences detected were almost identical (99.9%) to vv MDV detected in broiler chickens in Tunisia. Lower nt identities, 99.31% and 99.41%, were found for the GaAHV-2 *meq* gene detected in commercial turkeys in Italy (MN017102) and backyard turkeys in Turkey (OK322357), respectively. The mean number of four consecutive proline molecules within the Meq protein was shown to correlate significantly with the pathotype classification. The most virulent isolates showed the lowest number of such repeats, whereas the attenuated isolates showed the highest number of these repeats [12,24]. The molecular characterization of the *meq* gene, the main GaAHV-2 viral oncogene, in this study, showed molecular characteristics suggestive of the vv pathotype due to the presence of four PPPP repeats. Furthermore, a lack of 177 to 180 bp insertions, typically found in isolates of lower virulence [22,25], additionally supports the theory of oncogenic virulent strains detected in this study. However, for precise pathotyping, experimental inoculation is required, emphasizing that the pathogenicity tests described were performed in chickens, and the pathogenicity of the MDV strains detected could differ between chicken and turkey hosts.

Many studies assume that turkeys could contract the infection through direct or indirect contact with GaAHV-2-infected chickens [15]. The discovery of vv MDV strains correlates with the increased number of MD cases in turkeys and is one potential hypothesis that could explain the increase in MDV incidence in turkeys [35]. Moreover, recent detection of the GaAHV-2 partial DNA polymerase gene nt sequence in Ural owls in Slovenia raises the possibility that free-living birds should also be considered as a risk for GaAHV-2 infection [36]. However, even though most documented MD cases were reported in turkeys that were reared with or close to chickens [12,14], in the cases presented here, no other poultry species were present at the farms during the fattening cycle nor in the past. All three flocks have the same veterinarian. However, given the over 1-year gap between MD occurrences in each flock, it seems improbable that the MD cases observed are nosocomial infections stemming from the veterinarian. Furthermore, the veterinarian, who exclusively handles the care and treatment of these turkeys, works solely with commercial turkeys and not with any other poultry species. This eliminates the possibility of MDV infection from sources such as broiler chickens or layers. All three cases occurred in enclosed commercial turkey farms with relatively good biosecurity standards, which should prevent the introduction of the disease from potentially infected free-living birds. However, more detailed studies are needed to have a better understanding of the epidemiology of MD in turkeys.

## 5. Conclusions

This study presents the first clinical and pathological findings and sequence-based evidence for MD that occurred in three commercial turkey flocks in Slovenia between 2021 and 2023. The GaAHV-2 Meq protein detected was identical and most similar (99.9%) to the GaAHV-2 sequence detected in chickens in Tunisia. Based on phylogenetic analysis and the number of PPPP motifs within the Meq protein, MDV detected in Slovenia was suggestive of the vv pathotype. However, despite the identical detected sequences, clinical signs of illness varied between flocks, from no observed clinical signs to depression, lameness, weakness, and increased mortality. Although vaccination against MDV in turkeys showed its effectiveness in some cases, caution should be applied, especially considering that vaccinations against MDV in chickens have significantly contributed to the increase in virulence over the past decades. However, undoubtedly, the most important control measures are increased surveillance and enhanced biosecurity.

## Figures and Tables

**Table 1:** Primer sequence used for the molecular detection of pathogens.

Pathogen	Target Gene	Primer Name	Primer Sequence 5′–3′	Amplicon Size (bp)	Reference
**REV**	*rev* *envelope* gene	env-F	tcactctcgatggaaattgcag		[17]
		env-R	ccagtcctattgtctgcttccc	96	
		env probe	FAM-tagatgtcaactgctatgca-MGBNFQ		
	*ltr* gene	LTR-F	aggctcataaaccataaaaggaaatgt		
		LTR-R	cctttacaaccattggctcagtatg	119	
		LTR probe	FAM-acaaacacgagatcgaacta-MGBNFQ		
**LPDV**	*p31-ca* gene	LPDV F	atgaggacttgttagattggttac	457	[18]
		LPDV R	tgatggcgtcagggctatttg		
**MDV**	*meq* oncogene	EcoR-F	ggtgatataaagacgatagtcatg	1622	[16]
		EcoR-R	ctccaggagttccgaagtatgag		
		*meq*-pur-F1 *meq*-pur-R1	ccgcacactgattcctaggca ggattgtgcggggtggtaagc	506	This study
		*meq*-pur-F2 *meq*-pur-R2	ggagaagacgcagggagcag gaaggccccggagcgtag	406	
		*meq*-pur-F3 *meq*-pur-R3	cgctccacattgctccgg gggatcctcggtaagacgagc	622	
		*meq*-pur-F4 *meq*-pur-R4	ctacgctccggggctctg ggggcatagacgatgtgctgc	517	

**Table 2:** Details of the MDV-1 strains, retrieved from GenBank, that were used for the phylogenetic analysis.

GaHV-2 strain	Country	Year	Pathotype	Size of *Meq* (aa)	PPPPs	Host	Accession Number	References
CVI988	Netherlands	1969	att	398	7	NA	DQ530348	[21]
3004	Russia	NA	att	398	7	NA	EU032468	NA
CU-2	US	1970s	m	398	7	*Gallus gallus*	AY362708	[22]
MD70/13	Hungary	1970	v	339	5	*Gallus gallus*	MF431495	[23]
617A	US	1993	v	339	4	NA	AY362712	[22]
04CRE	Australia	2004	v	398	5	NA	EF523773	[24]
JM/102w	US	NA	v	399	7		DQ534539	[25]
Md5	US	1977	vv	339	4	NA	AF243438	[26]
C12/130	UK	1992	vv	339	5	chicken	FJ436096	[27]
02LAR	Australia	2002	vv	398	5	chicken	EF523772	[24]
RB1B	US	NA	vv	339	5	NA	AY243332	[22]
New	US	1999	vv+	339	2	NA	AY362719	[22]
W	US	1999	vv+	339	4	NA	AY362723	[22]
ATE2539	Hungary	2000	vv+	339	5	*Gallus gallus*	MF431493	[23]
TK	US	NA	vv+	339	2	chicken	AY362721	[22]
Italy/Ck/1083/18	Italy	2018	NA (vv/vv+)	339	4	commercial chicken	MK855066	[12]
Italy/Turkey/601/16	Italy	2016	NA (vv/vv+)	339	4	meat-type turkey	MN017102	[12]
Italy/Ck/850/17	Italy	2017	NA (vv/vv+)	339	5	backyard chicken	MK139674	[16]
TN1013/16	Tunisia	2016	NA (vv/vv+)	339	4	*Gallus gallus*	MK041219	[28]
TN1014/16	Tunisia	2016	NA (vv/vv+)	339	4	*Gallus gallus*	KY113150	[28]
Italy/Ck/847/17	Italy	2017	NA (vv/vv+)	418	10	backyard chicken	MK139672	[16]
MDV-1/TR21/turkey	Turkey	2021	NA (vv/vv+)	339	4	backyard turkey	OK322357	[14]
G2	China	2002	NA	339	4	chicken	AF493556	[29]

NA = not available; the strain was not subjected to the in vivo pathotyping test. Text in brackets = pathotype according to the authors’ suggestion.

## Data Availability

Sequence data obtained in this study were deposited in the NCBI database under accession numbers OQ868520, OQ868521, and OR490416.
